# Effects of 660-nm LED photobiomodulation on drebrin expression pattern and astrocyte migration

**DOI:** 10.1038/s41598-023-33469-5

**Published:** 2023-04-17

**Authors:** Sung Ryeong Yoon, So-Young Chang, Min Young Lee, Jin-Chul Ahn

**Affiliations:** 1 Department of Medical Science, Graduate School of Medicine, Dankook University, Cheonan, 31116 Republic of Korea; 2 Medical Laser Research Center, College of Medicine, Dankook University, Cheonan, 31116 Republic of Korea; 3 Department of Otolaryngology-Head & Neck Surgery, College of Medicine, Dankook University, Cheonan, 31116 Republic of Korea; 4 Beckman Laser Institute Korea, College of Medicine, Dankook University, Cheonan, 31116 Republic of Korea

**Keywords:** Biophysics, Cell biology, Applied optics, Cellular neuroscience

## Abstract

Photobiomodulation (PBM) is a therapeutic tool that uses red or near-infrared light in medical applications. It’s applications in both central (CNS) and peripheral nervous system (PNS) are widely studied. Among glial cells, astrocytes are known to be activated in injured or damaged brains. Astrocytic cell migration is crucial for maintaining homeostasis in the brain. Our previous study showed that PBM led to astrocyte proliferation and differentiation, but the effects on migration has not been investigated. The aim of this study was to evaluate the effect of PBM on astrocyte migration, drebrin (DBN) expression and cytoplasmic morphology using primary cultured rat astrocyte. We applied a 660-nm light-emitting diode (LED) with fluence of 6, 12 and 18 J/cm^2^. PBM effects on astrocyte migration were analyzed by two different migration assays (scratch assay and transwell assay). We used immunofluorescence microscopy for visualizing DBN and glial-fibrillary acidic protein (GFAP) and analysis of DBN expression and astrocyte cytoplasmic morphology. Both scratch assay and transwell assay showed significant difference in astrocyte migration following PBM irradiation. With these specific fluence conditions, differences in DBN expression and cell morphology were revealed. PBM could increase the astrocyte migration by altering the cell morphology and DBN expression pattern.

## Introduction

The brain is composed of neurons and glial cells. Glial cells include microglia, astrocytes, and oligodendrocytes, which support neurons in inflammation clearance and maintenance. Because glial cells do not contain axons or dendrites, they do not participate in electrical synaptic signaling^1,2^. Astrocytes are the most numerous glial cells in the brain; they perform various roles, including neuron support, waste removal from synapses, and phagocytosis^3^. Astrocytes also protect neurons during brain injury. Reactivity, a defensive astroglial reaction, is increased by injury. Reactive astrogliosis occurs under many pathological conditions such as ischemic damage, neurodegeneration, and neuroinflammation^4,5^. Astrocytes are quiescent under normal conditions but are activated in injured or damaged adult brains^6,7^. Once brain is injured, astrogliosis occurs as a defense mechanism and plays a role in regulating inflammation and wound healing^5,8^. This ‘reactive’ gliosis can be observed in astrocyte morphology and function. In terms of morphology, the soma or protrusion part of cell is enlarged, polarization occurs, and extension that stretches the processes occurs^7^. These features could be the indirect evidence of cell migration to the injury site. After migration, scar formation process occurs and prevents further damage to non-injured areas^6,8–10^. Therefore, astrocytic cell migration is crucial during injury and for maintaining homeostasis in the brain.

Photobiomodulation (PBM) includes a low level light therapy that uses red or near-infrared light in medical applications such as wound healing, tissue repair, and inflammation relief^11–13^. PBM also promotes cell proliferation and stem cell differentiation^14^. Because PBM is non-invasive, it can be used for direct treatment by irradiating the target area^13,15^. PBM with specific wavelengths such as 660, 810-nm can penetrate the human brain^16^. Its application in brain diseases such as traumatic brain injury (TBI) and neurodegenerative and psychiatric disorders is being increasingly studied^17^. One recent study showed that hemoglobin in the brain and PBM mediate neuroprotective effects in an Alzheimer’s disease-like transgenic rat model^18^, another study showed that PBM attenuates epileptic excitotoxicity, thereby improving the synapse and cognitive function^19^. In addition, recent review suggested that PBM can induce neuroprotective effects on mitochondrial dysfunction in brain diseases such as Alzheimer’s disease, TBI, depression, and stroke^20^.

Cytochrome C oxidase (CCO) regulation is the most well-known mechanism triggered by PBM. In the mitochondrial respiratory chain, CCO is photoactivated by absorbing photons, enhancing the mitochondrial membrane potential (MMP), producing ATP, and finally triggering diverse downstream signals^13,17^. However, the precise mechanisms of CCO action in other processes remain elusive, and there is no evidence of correlation between red light-emitting diode (LED) light and astrocytes. Our previous study showed that PBM led to astrocyte proliferation and differentiation, but the effects on migration have not been investigated^21^. The objective of the present study was to explore PBM effects other than MMP-induced downstream signaling on astrocytes, particularly with respect to astrocyte migration.

Diverse events occur during astrocyte migration, including polarization and the regulation of actin cytoskeletal elements; these processes lead to morphological and functional changes in astrocytes^22^. Astrocytes near injury sites are polarized, and reactive astrogliosis triggers process extension^23^. These processes require the integration of many proteins related to cell migration^24^. The molecular mechanism controlling cytoskeletal organization was recently discovered^25^. Drebrin (DBN) is an actin-binding protein and cytoskeletal regulator that plays a pivotal role during astrocyte migration. DBN stabilizes actin filaments by preventing other actin binding-proteins from binding to them^26^. DBN has mainly been studied in neurons, whereas fewer studies have examined the relationship between DBN and astrocytes^27^, most of which are related to the gap junction protein complex, in which DBN binds to connexin-43 (Cx43), which is abundantly expressed in astrocytes and is crucial for maintaining the Cx43-related gap junction^28^. A recent study showed that DBN is an injury-specific actin regulator that enables astrocyte polarization. Ultimately, DBN is involved in scar formation and wound healing in the brain^25^.

Therefore, in the present study, we examined astrocyte migration with PBM using several migration assays and performed epifluorescence analysis of DBN expression and astrocyte cytoplasmic morphology.

## Results

### Astrocyte migration alteration under 660-nm LED light via the scratch assay

**Figure 1:**
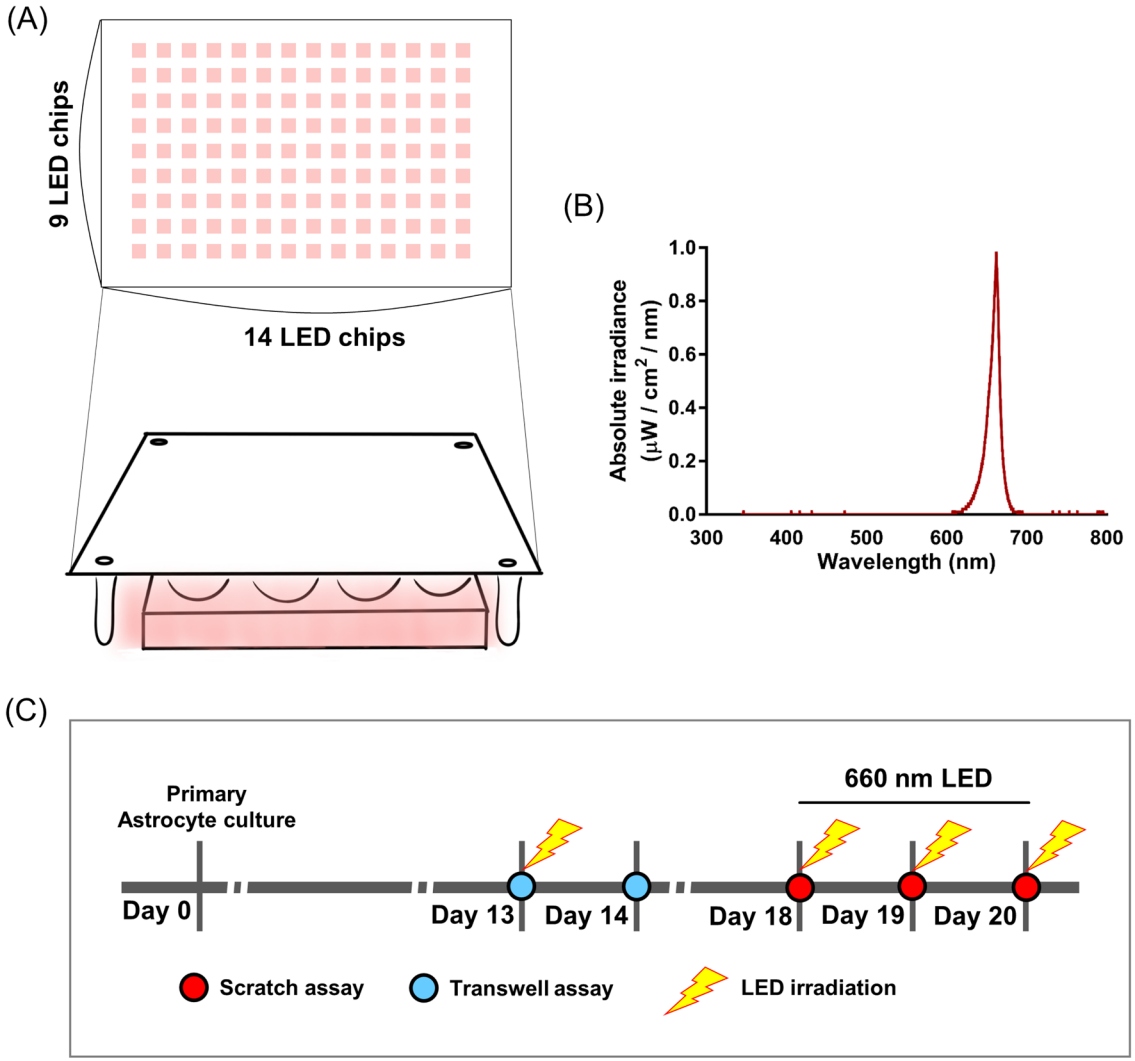
A 660-nm LED irradiation method and experimental schedule for each assays (**A**) Composition of LEDs and LED irradiation. (**B**) A graph showing 660-nm parameter delivered. (**C**) Timing of the transwell (blue circles) and scratch (red circles) assays performed in this study. The transwell assay was completed at 14 days in vitro (DIV); light-emitting diode (LED) irradiation was performed 1 day before the assay. The scratch assay was performed at 18 DIV; LED irradiation was applied with fluence of 6, 12 and 18 J/cm^2^ per day for three consecutive days.

**Figure 2:**
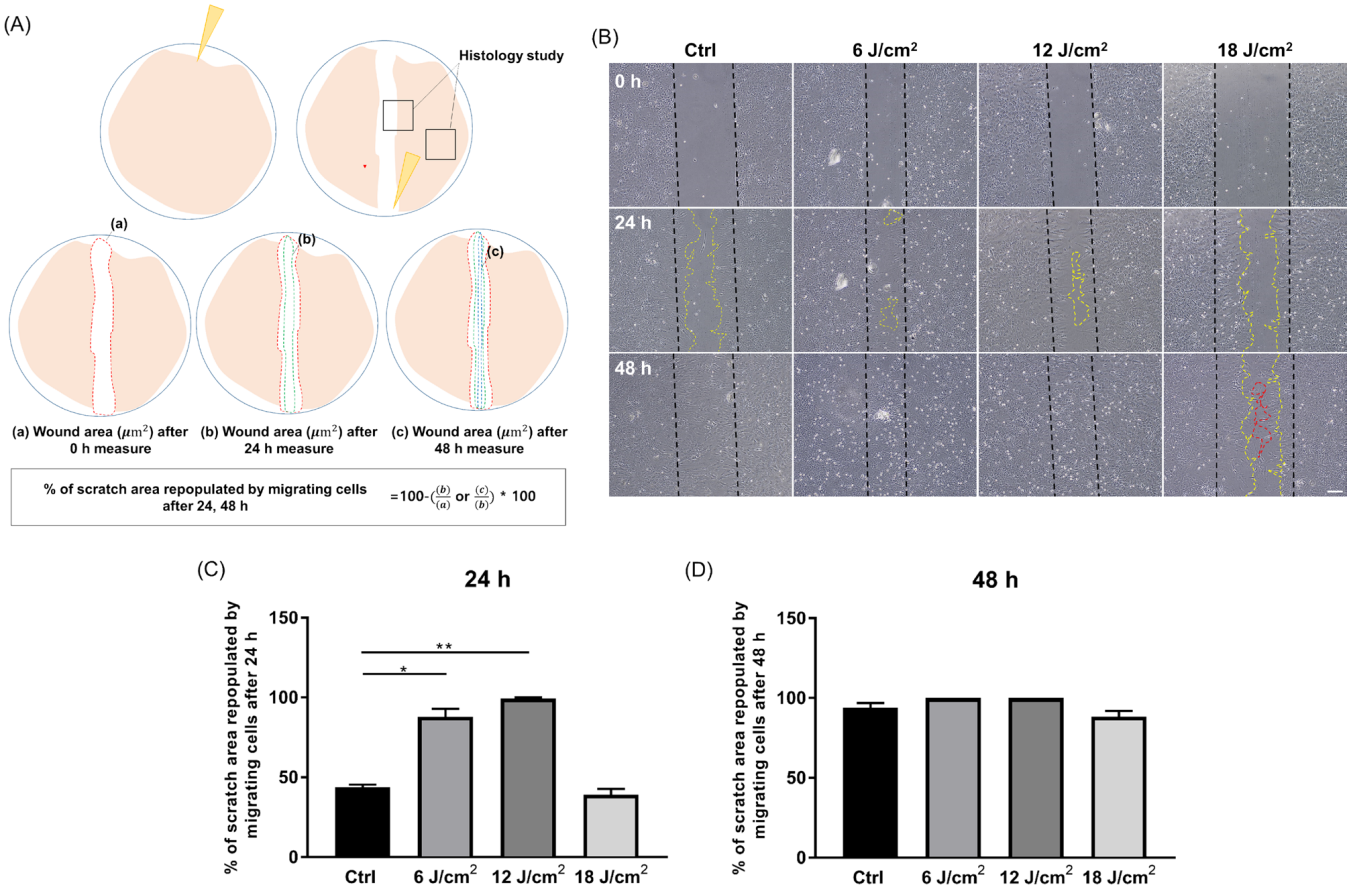
Scratch assay results. (**A**) At 18 DIV, the astrocyte monolayer was scratched in one direction using a yellow pipette tip (red arrow). Black squares indicate histological regions of interest. Red, green, and blue dotted lines indicate the expanded wound areas (μm^2^), respectively, after wounding. % of repopulated scratch area was calculated using the equation shown. (**B**) Representative images of scratches. Black, yellow and red dotted lines indicate the initial and expanded wound areas. Scale bar = 100 μm. (**C**, **D**) Graphs showing % of scratch area repopulated by migrating cells after 24, 48 h, respectively (*P < 0.05; **P < 0.01).

The scratch assay is commonly used to evaluate cell migration. We used a 200-μm yellow pipette tip to make vertical scratches on a monolayer astrocyte culture, irradiated the cells under a 660-nm LED (6, 12, and 18 J/cm^2^). After 0, 24, and 48 h, images were captured using a microscope (Figs. 1 and 2A). The wound areas (μm^2^) for the experimental groups are shown in Fig. 2B. At 24 h after the scratch assay, the wound areas were smaller in the 660-nm LED exposure group (6 and 12 J/cm^2^) than in the control and 18 J/cm^2^ group, and the differences were significant (Kruskal–Wallis test and post hoc: *P < 0.05; **P < 0.01; Fig. 2B,C). At 48 h after the scratch test, there was no difference between the control and 660-nm LED exposure groups (Kruskal–Wallis test and post hoc: *P* > 0.05; Fig. 2D), because the gaps were filled by cells in all groups.

**Figure 3:**
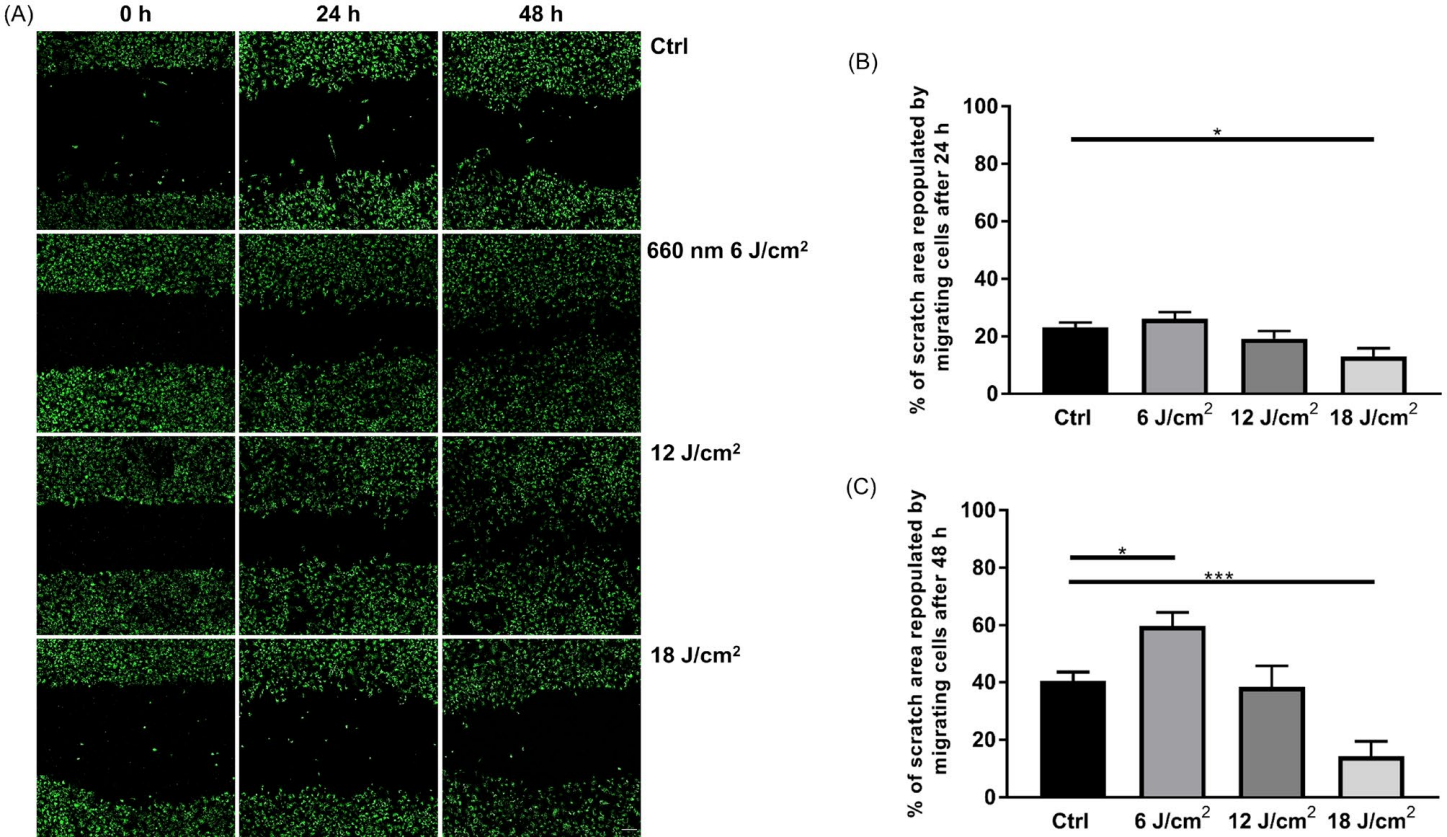
Cytoplasmic membrane dye stained cell migration tracking. (**A**) Representative images of dye stained live cell migration within each group at specific time points. Scale bar = 100 μm. (**B**, **C**) % of repopulation of migrating cells after 24, 48 h, respectively (*P < 0.05; ***P < 0.001).

To further investigate 660-nm LED effects, we tracked astrocytic migration with fluorescence live cell staining. We treated cytoplasmic membrane dyes and imaged at a specific time point (0, 24, and 48 h after scratch; Fig. 3A). There was significant difference between control and 660-nm LED (6 J/cm^2^) group 48 h after scratch assay (one-way ANOVA; control vs. 660-nm LED, n = 15 vs. 6; *P < 0.05; ***P < 0.001; Fig. 3A,C). Along with the prior results shown in Fig. 2, these results confirm the enhanced migration of astrocyte by PBM and is pointing out the most optimal LED irradiation fluence which is 6 J/cm^2^. There is mismatch between light microscopic evaluation and fluorescence assessment. This difference would be possibly due to the visible cytoplasmic extension in light microscope which is not stained by green fluorescence in current protocol. In case of 660-nm LED irradiation with 18 J/cm^2^, % of repopulated scratch area was significantly decreased both 24, 48 h after scratch (Kruskal–Wallis test for 24 h; one-way ANOVA for 48 h; control vs. 660-nm LED, n = 15 vs. 6; *P < 0.05; ***P < 0.001; Fig. 3B,C). This result implicates that there is an optimal fluence of 660-nm LED irradiation, and cells are not affected by PBM outside than optimal conditions.

### DBN expression pattern changes under 660-nm LED exposure

**Figure 4:**
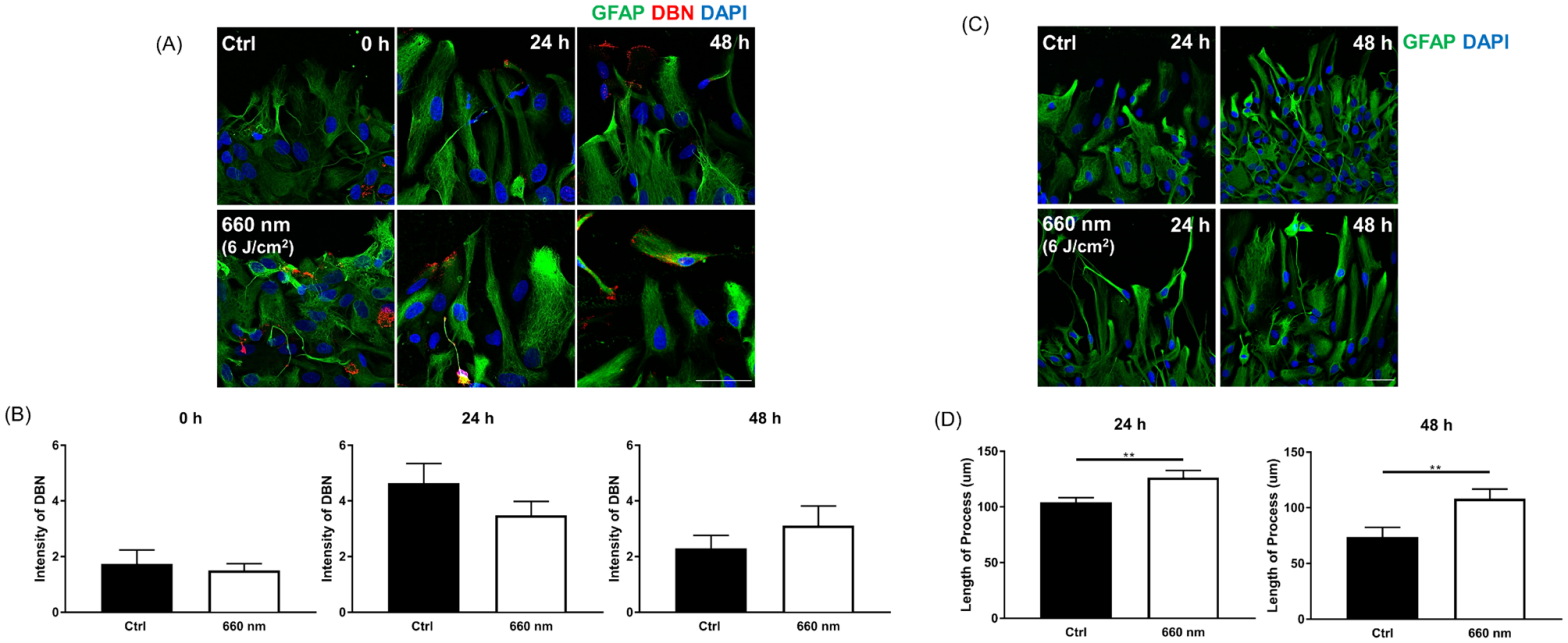
Comparison of astrocyte morphology between the control and 660-nm LED (6 J/cm^2^) exposure groups after wounding (**A**) Representative images show immunofluorescent staining at 0, 24, and 48 h after wounding. Red: drebrin (DBN), an actin-binding protein; green, glial fibrillary acidic protein (GFAP; astrocytes). Scale bar = 50 μm. (**B**) DBN intensity at 0, 24, and 48 h after wounding; no significant differences were detected between control and 660-nm LED exposure groups (*P* > 0.05; control vs. LED, n = 12 vs. 10 at 0 h; n = 22 vs. 19 at 24 h; n = 18 vs. 23 at 48 h). (**C**) Representative image of cytoplasm process evaluation using fluorescence images. Scale bar = 50 μm. (**D**) Analysis of cytoplasm processes for astrocytes adjacent to injury sites at 24 and 48 h after wounding. At both 24 and 48 h, cytoplasm processes were significantly longer in the 660-nm LED exposure group than in the control (*P* < 0.01; control vs. LED, n = 33 vs. 33 at 24 h; n = 24 vs. 27 at 48 h).

We confirmed that 660-nm LED (6 J/cm^2^) promotes astrocytic migration in a scratch assay. Thus, we searched for candidate molecule mediating the effect by 660-nm LED exposure. A previous study showed that astrocytes express DBN in brain injury, where it regulates cell morphology through microtubules. DBN is crucial for scar formation and astrocyte reactivity^25^ and protects neurons in injured brain tissue exposed to increased oxidative stress^29^. To evaluate DBN expression after the scratch assay, immunofluorescence staining of GFAP and DBN was performed at 0, 24, and 48 h after scratching (Fig. 4A). DBN intensity did not differ between the control and 660-nm LED (6 J/cm^2^) irradiation groups at any time points (Fig. 4B). To assess the reactivity of astrocytes near the scratch, GFAP intensity was observed and astrocyte cytoplasm processes were measured. We observed high GFAP intensity (homogeneous expression) within the scratched area, suggesting that astrocyte reactivity was increased by injury (Fig. 4A,C). Cytoplasm processes were significantly longer under 660-nm LED (6 J/cm^2^) exposure than in the control (unpaired *t*-test; control vs. LED, *P* = 0.0049, n = 33 vs. 33, (24 h); *P* = 0.0068, n = 24 vs. 27, (48 h); Fig. 4C,D) This significant increase in cytoplasmic extension could be an alternative evidence of that 660-nm LED induced facilitation of astrocyte migration.

**Figure 5:**
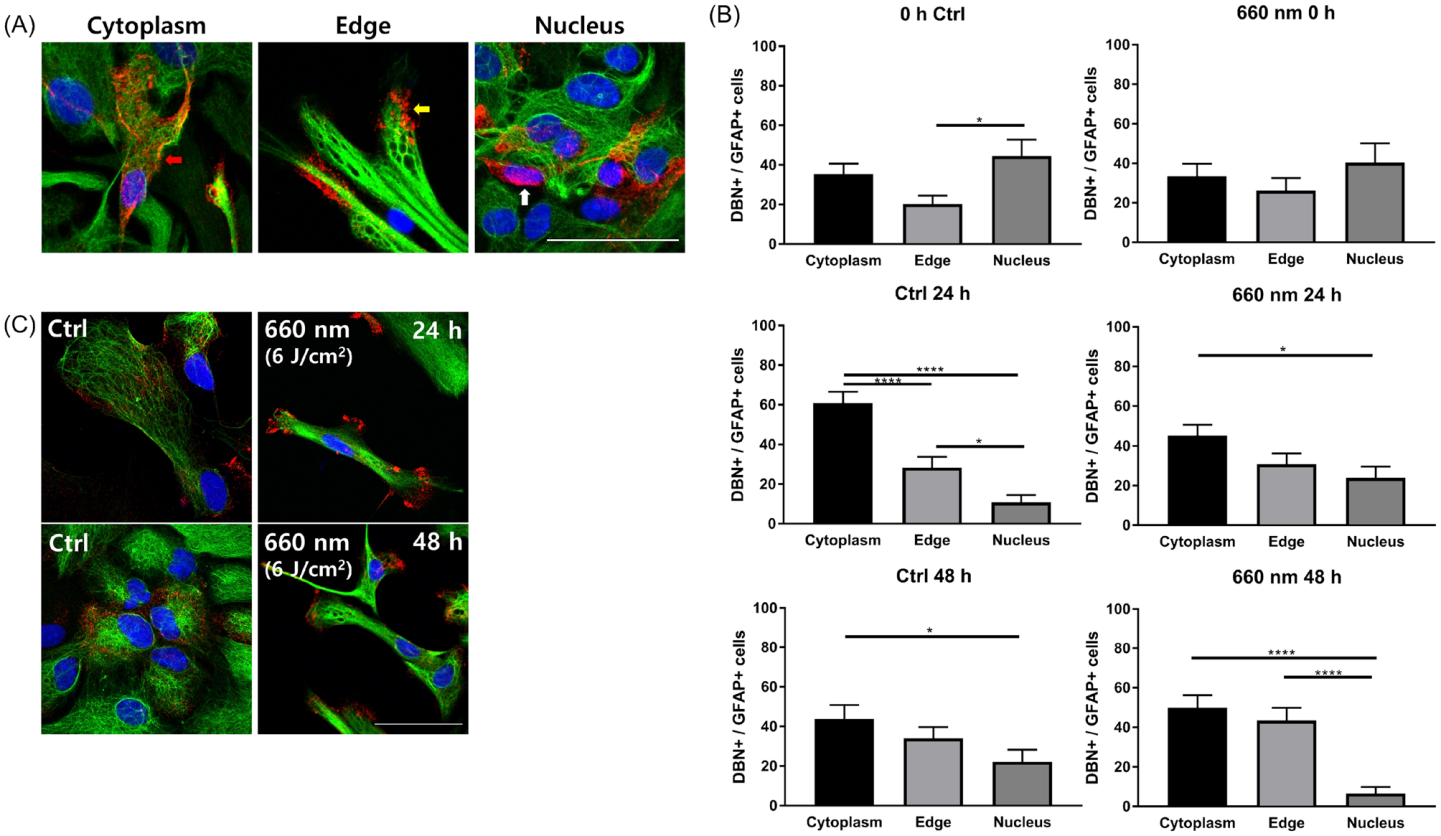
Differences in DBN expression patterns following 660-nm LED (6 J/cm^2^) exposure. (**A**) DBN expression patterns were divided into three categories: cytoplasm (red arrow), edge (yellow arrow), and nucleus (white arrow). Scale bar = 50 μm. (**B**) Ratios of DBN-positive cells to GFAP-positive cells at 0, 24, and 48 h after wounding (*P* < 0.0001; control vs. LED, n = 12 vs. 10 at 0 h; n = 22 vs. 20 at 24 h; n = 17 vs. 22 at 48 h). (**C**) Representative images corresponding to (**B**). Scale bar = 50 μm. In (**B**, **C**), cytoplasm DBN distribution was dominant after wounding; we speculate a decrease in DBN distribution among nucleus. At 48 h after wounding, the LED exposure group showed a marked increase in edge distribution compared with the control.

To analyze DBN expression patterns, we counted DBN- and GFAP-positive cells and categorized the cells according to their expression patterns in cytoplasm, edge, and nucleus ROIs (Fig. 5A). Distribution patterns of DBN expression location changed over time. At 0 h after scratching, both the control and 660-nm LED (6 J/cm^2^) irradiation groups showed similar expression patterns among locations, with the lowest rate of DBN expression in edge ROIs. At 24 h after scratching, increases of cytoplasm expression of DBN were noted in both groups. At 48 h after scratch, relatively increased expressions in edge location were suspected. As to the comparison of expression pattern at each time point in each group, in the control group at 0 h, DBN expression was significantly higher in nucleus than at the edge of astrocytes (one-way ANOVA and Tukey’s test; edge vs. nucleus, *P* = 0.0255; Fig. 5B). At 24 h, the control and 660-nm LED (6 J/cm^2^) irradiation groups showed similar patterns, with the lowest DBN expression observed in astrocyte nucleus and the highest in the cytoplasm (one-way ANOVA and Tukey’s test; control: cytoplasm vs. edge and cytoplasm vs. nucleus, *P* < 0.0001, edge vs. nucleus, *P* = 0.0445; 660-nm LED: cytoplasm vs. nucleus, *P* = 0.0209; Fig. 5B). Differences in expression between groups became smaller following 660-nm LED (6 J/cm^2^) irradiation at 24 h. After 48 h, expression patterns were similar to those at 24 h; however, the 660-nm LED (6 J/cm^2^) irradiation group showed a dramatic decrease in DBN expression in the nucleus (Fig. 5B,C). In 660-nm LED-irradiated astrocytes, DBN expression was significantly lower in the nucleus than in the cytoplasm and edges (one-way ANOVA and Tukey’s test; 660-nm LED: cytoplasm vs. nucleus, edge vs. nucleus, *P* < 0.05, Fig. 5B). Edge DBN expression increased substantially, suggesting that the location of DBN expression may alter astrocyte migration following 660-nm LED exposure.

### Astrocyte migration alteration under 660-nm LED light via the transwell assay

**Figure 6:**
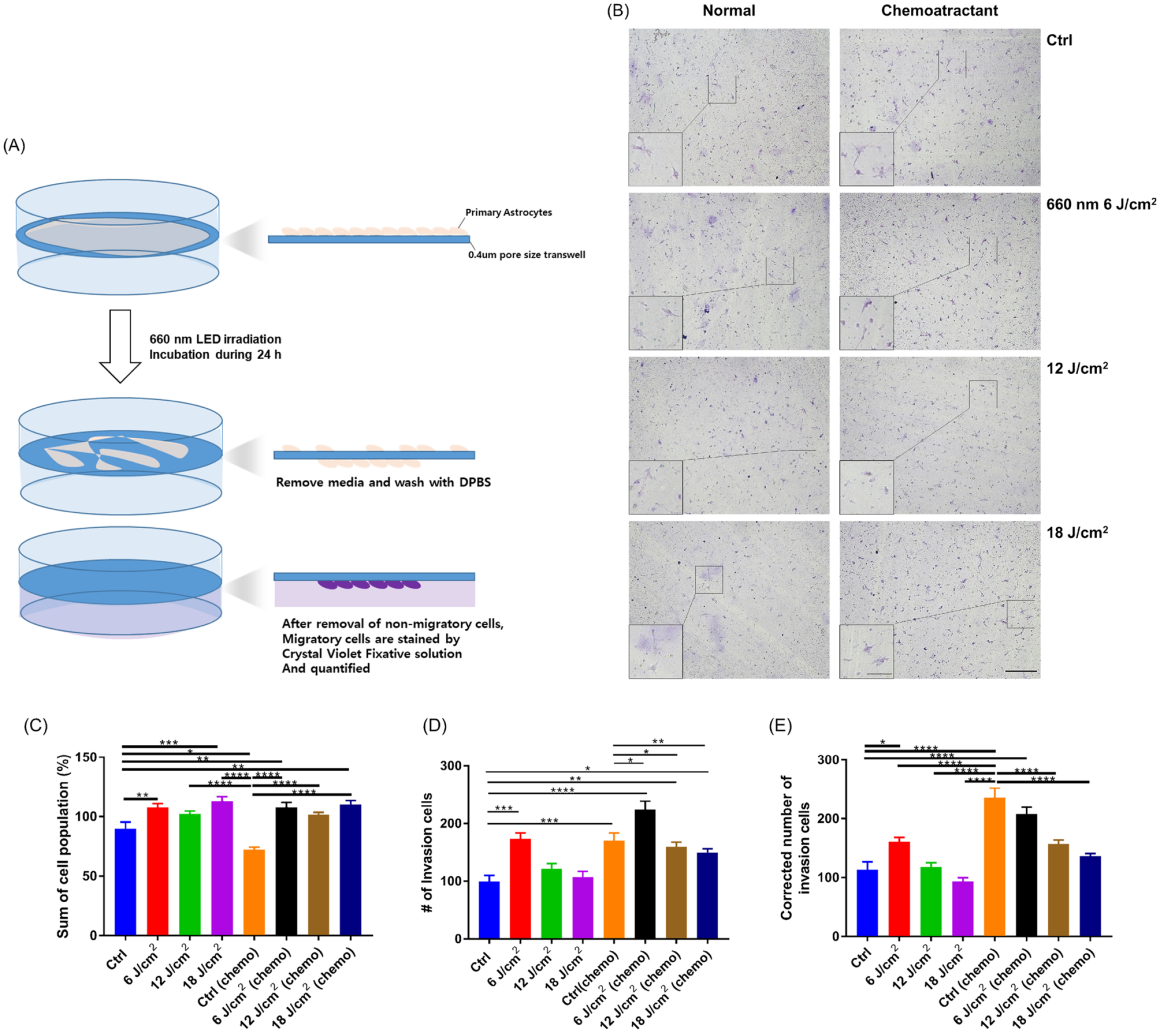
Transwell assay results. (**A**) On DIV13, astrocytes were subcultured and seeded on transwell plates, and then irradiated at 660 nm. After 24 h, media were removed and cells were washed with Dulbecco’s phosphate-buffered saline (DPBS). Crystal violet fixative solution was applied. Non-migratory cells were removed and migratory cells were stained with the solution and imaged using differential interference contrast microscopy. The number of cells was manually counted. (**B**) Representative images showing invasion cells under the transwell [Scale bar: 100 μm; 25 μm (magnification images)]. (**C**–**E**) Graphs showed the significant alteration of cell invasion following 660-nm LED exposure (*P < 0.05; **P < 0.01; ***P < 0.001; ****P < 0.0001; control vs. LED, n = 9 vs. 9). (**C**) A graph showed the sum of cell population (%) of both sides (on and under) the transwell. (**D**) A graph showed the number of invasion cells without the correction. (**E**) A graph showed the corrected number of invasion cells [number of invasion cells/sum of cell population (%)].

To confirm the results of the scratch assay, which is relatively invasive as the pipette tip can damage cells near the scratch, we also performed a non-invasive transwell assay. To test uninjured astrocyte migration, astrocytes were seeded onto a transwell membrane and then irradiated with 660-nm LED light (6, 12, and 18 J/cm^2^) with or without chemoattractant (10% FBS). After 1 day, crystal violet staining was performed; the results are shown in Fig. 6. The cell concentration (summary of both on and under the transwell) was automatically counted through Olympus microscope software program and compared among groups, which showed statistical increase of cell population in LED groups (except 12 J/cm^2^ without chemoattractant) compared to control with or without chemoattractant (one-way ANOVA; control vs. 660-nm LED, n = 9 vs. 9; *P < 0.05; **P < 0.01; ***P < 0.001; ****P < 0.0001; Fig. 6C). As to the invasion cells, which refers to migrated cells (under the transwell membrane), significantly increased invasion cells (astrocyte migration) was observed in the 660-nm LED exposure (6 J/cm^2^) treatment group compared to the control. But 660-nm exposure with 12 and 18 J/cm^2^ was similar or decreased compared to the control and not significant (one-way ANOVA; control vs. 660-nm LED, n = 9 vs. 9; *P < 0.05; **P < 0.01; ***P < 0.001; ****P < 0.0001; Fig. 6D). As expected, chemoatractant conditions showed higher migration than normal conditions (Fig. 6B,D,E) (Fig. S1).

To avoid criticism of the confounding effect of cell proliferation increased by 660-nm LED, the number of invasion cells were corrected based on the measurement of overall cell concentration [corrected number of invasion cells: number of invasion cells / sum of cell population (%)]. After correction of invasion cell, there was no alteration of significance as to groups without chemoattractant, showing statistically increased number only in 6 J/cm^2^ (660-nm LED exposure) group compared to control. However, tendency of alteration in corrected invasion cells by 660-nm LED was different in chemoattractant conditions, showing gradual decrease of corrected invasion cells over LED exposure time. In both corrected and uncorrected conditions, cells followed by 660-nm LED exposure (12 and 18 J/cm^2^) were significantly decreased compared to the control (one-way ANOVA; control vs. 660-nm LED, n = 9 vs. 9; *P < 0.05; **P < 0.01; ***P < 0.001; ****P < 0.0001; Fig. 6D,E). But statistical increase of invasion cells in 6 J/cm^2^ LED group observed in uncorrected condition was not observed after correction. Altogether, consistent with the results of the scratch assay, this result confirmed that astrocyte migration was enhanced by 660-nm LED irradiation at specific condition (6 J/cm^2^).

## Discussion

This study is the first to examine the effects of PBM on astrocyte migration. We performed two types of astrocyte migration assays; both assays showed significant differences in astrocyte migration. Furthermore, differences in DBN expression and cell morphology following PBM application were revealed. The reason for two different ways of evaluating the migration was to reduce the possible confounding effects of cellular trauma. Once injury occurs, astrocytic migration follows as astrocytes become reactive. In the scratch assay, cellular injury may have occurred due to mechanical trauma by the pipette tip; such injury can cause cell trauma, altering their responsiveness to PBM irradiation.

There is possibility that early scar formation by enhanced astrocyte migration can limit the regeneration of axons and synapses. Decreased axonal regeneration after scar formation has been reported numerously^8–10^. PBM induced early scar formation could reduce the natural neural regeneration process and inhibit natural recovery of brain. Nevertheless, aberrant axonal growth might be also problematic to recovery. For example, synkinesis which is wrong connection of damaged axons could be more difficult to manage and cause complications to patients. Moreover, another study showed that enhanced astrocyte migration was accompanied by accelerated neural wound healing after inducing oxygen glucose deprivation in vitro^30^. So, there is still controversies regarding the neural wound healing and scar formation in terms of astrocyte recruitment. But it is quite clear that early migration of astrocyte would prevent expansion of damage process as mentioned in introduction. The promotion of glial scar formation can help wound healing by preventing the migration of other inflammatory cells and secondary change at the damaged area. Thereby protecting non-injured tissue from secondary damage^23,31^. Previous study showed that, in focal brain injury mouse model, reactive astrocytes formed a protective barrier and attenuated the toxic environment for neuronal survival^32^. In our study, although we did not confirm in neuron and astrocyte co-culture and in vivo study, it is expected that rapid promotion of migration by PBM would rapidly reach to the protective effect against secondary damage.

In the present study, intensity of GFAP was increased nearby injured site. Expression of GFAP is one of the markers for astrocytic activity. This ‘active’ astrocyte could be divided into scar-forming astrocyte and reactive astrocyte. Previous studies showed that astrogliosis and scar formation did not occur, and defense mechanisms did not work properly in the GFAP KO model^31,33^. Reactive astrocyte could be divided into two subtypes: A1 and A2 astrocytes. A1 astrocytes result in neuronal damage and death and A2 astrocytes acts otherwise performing CNS recovery and repair^34,35^. Therefore, not all the reactive astrocytes are beneficial. Currently, it is not clear which cell type these increased GFAP cells would be. But considering that this process is following the scratch, it is highly probable that purpose of this GFAP increase would be scar-formation.

In this study, only 6 J/cm^2^ dose LED irradiation was applied to analyze DBN expression. The determination of the LED dosage was carefully confirmed by scratch and transwell assay. Taken the outcome of each scratch and transwell assay together, a dose of 6 J/cm^2^ LED irradiation results in significantly increased migration of astrocytes compare to 12 and 18 J/cm^2^. One of the interesting findings is that 18 J/cm^2^ LED irradiation rather deteriorated cells. The reason for this negative result in higher dosage could be biphasic effect of PBM. Previous studies showed that cells require optimal irradiation conditions to activate biological reaction^36^. Neither cells react at very low dose nor very high dose. Mediators of cell signaling by low energy density PBM can be induced to exert biomodulatory effects, while beneficial mediators are decreased by high energy density PBM, thereby potentially increasing detrimental mediators^37,38^.

Our previous study showed that cell proliferation markers such as Ki67 were increased by 660-nm LED exposure^21^. Therefore, we cannot neglect the possibility that differences shown by the transwell assay were caused by increased proliferation rather than migration. However, according to a cell viability assay in our previous study, varying PBM intensity did not significantly increase cell viability. Furthermore, in the present study, we have measured the cell concentrations and corrected migration assay using these measurements showed significant increase in the specific LED parameter as well. Thus, our results likely indicate increased migration rather than proliferation.

We detected no significant difference in DBN expression intensity in response to PBM exposure at any time point after injury. Considering the fact that DBN expression is influenced by injury^25^, we investigated a bit further and performed an analysis of the DBN expression location within the astrocyte. Plausible explanation for differences of DBN expression locations would be the translocation of DBN from nucleus to cytoplasm (or to edge of cytoplasm in case of possible migration) after activation. Indeed, there were expression pattern changes over time as mentioned in the result. Even distribution at beginning, robust increase of cytoplasm expression at 24 h (after trauma), and subtle increased expression in edge location at 48 h were observed. In the present study, the ratio of the DBN expression distribution was influenced by PBM exposure. At 24 h after injury, DBN expression in the cytoplasm was decreased by PBM and became more evenly distributed at each location. These results imply that astrocyte activation (nucleus to cytoplasm translocation) was down regulated by PBM. Since this time point is relatively short duration after trauma (scratch), it is feasible to assume that this trauma induced reduction of secondary process. At 48 h after scratch, relative longer duration after scratch, cell edges showed significantly higher DBN expression under PBM exposure. Along with the elongation of cellular processes, these results might indicate that DBN is activated to construct the cytoplasmic structures to elongate the cellular sprouting which could lead to further migration of astrocytes. Several mismatches among studies should be clarified. Nevertheless, most of the results are providing evidence of alteration of DBN, cell morphology and migration itself after PBM.

As discussed, this is the first study to seek the migratory enhancement of astrocytes by PBM. Therefore, to elucidate the detailed mechanism in after coming studies, suggesting the limitations of current study would be a crucial step. First, it is necessary to examine the molecular mechanism underlying these results to determine whether reactive astrocyte migration activity is inhibited by PBM, as reported in a previous study on signaling pathways related to inflammation. Further studies should explore how astrocyte migration is altered in the presence of neurons and neuronal injury, both in vitro and in vivo. It is also necessary to determine how the expression of cytoskeletal- and migration-related proteins changes in relation to DBN expression pattern changes. Finally, in this study, the effects of PBM on cell migration were studied only at a wavelength of 660 nm; future studies should examine changes in cell migration at other wavelengths.

## Materials and methods

### Ethics declarations

All experiments were performed in accordance with protocols approved by the Institutional Animal Care and Use Committee of Dankook University, Republic of Korea and with the relevant guidelines and regulations. The authors complied with the ARRIVE guidelines.

### Primary cell culture

Cultured astrocytes were prepared as described previously^39^, but with minor modifications. Animals were purchased from Nara Biotech (Seoul, Republic of Korea) and euthanized by Urethane (Daejung, Gyeonggi, Republic of Korea) dissolved in tertiary distilled water (12.5%, 0.8 ml/100 g). Briefly, brains were extracted from Sprague Dawley rats at embryonic day 17. Five pregnant animals were sacrificed and the number of litters was about 12 for each. Cortices were isolated from extracted brain tissues and incubated in a water bath at 37 °C with 0.25% trypsin for 20 min and then gently triturated. Dissociated cells were counted and inoculated into culture dishes (4 × 10^6^ cells/60-mm dish) coated with 0.04% polyethylenimine (PEI; Sigma-Aldrich, St. Louis, MO, USA) and then incubated at 37 °C in a 5% CO_2_ humidified incubator. The culture media consisted of Dulbecco’s modified Eagle’s medium (Gibco, Thermo Fisher Scientific, Waltham, MA, USA) supplemented with 10% fetal bovine serum (Corning Inc., Corning, NY, USA), 5000 U/mL penicillin, and 5000 U μg/mL streptomycin (Corning Inc.). On day 6 of culture, the culture dish was shaken at 110 rpm for 6 h and then treated with 0.25% trypsin to suspend the cells. Suspended cells were cultured by the redistribution of 2 × 10^5^ cells per well into 12-well plates containing PEI-treated coverslips.

### Photobiomodulation under 660-nm LEDs

To investigate the effects of red light on astrocyte migration, we applied a 660-nm LED (WONTECH Co. Ltd., Seoul, Republic of Korea; Fig. 1B) with fluence of 6, 12, and 18 J/cm^2^. The LEDs (n = 126; 14 × 9) were arranged on a printed circuit board (14.6 × 21.0 cm). LED light was delivered from above of cell culture plates (Fig. 1A). LED irradiation proceeded as shown in the Fig. 1C. One day after 6, 12 and 18 J/cm^2^ LED irradiation of astrocytes, we performed a transwell assay in the dark. In a scratch assay, LED irradiation was applied with fluence of 6, 12, and 18 J/cm^2^ per day for three consecutive days (Fig. 1C). In each experiment, we measured the LED irradiance (mW/cm^2^) using a power meter (VEGA, Ophir PD, Jerusalem, Israel) and power detector (PD300-TP-ROHS, Ophir PD, Jerusalem, Israel) located under the middle of the LED; measurements were performed in the dark.

### Cell migration analysis

#### Scratch assay

At 18 days in vitro (DIV), cells were scratched in one direction using a 200-μm yellow pipette tip (Axygen, Union City, CA, USA) and then irradiated with a 660-nm LED. To calculate % of repopulated scratch area, cells were imaged daily by interference contrast microscopy (ICM; Olympus, Tokyo, Japan) for 3 days. For immunofluorescence analysis, cells were fixed with 100% MeOH at 0, 24, and 48 h after the scratch assay. To track live cell migration as an another method, CellBrite® Green cytoplasmic membrane dyes (biotium, Fremont, CA, USA) were treated right after scratch.

#### Transwell assay

One day before the transwell assay, cells were seeded into a transwell plate (0.4 μm pore size; Costar, Cambridge, MA, USA) and irradiated with a 660-nm LED. After 24 h, the media were removed and cells were washed with Dulbecco’s phosphate-buffered saline (Corning Inc.). Non-migratory cells were removed using cotton swabs, and migratory cells were stained with crystal violet fixative solution containing crystal violet powder (Sigma-Aldrich), 10% neutral buffered formalin (BBC Biochemical, Mount Vernon, WA, USA), 10 × phosphate-buffered saline (PBS; Biosesang, Seoul, Republic of Korea), 100% MeOH, and dH_2_O, and then the solution was removed. Stained cells were washed with dH_2_O and dried in air. The stained cells were observed by ICM and counted manually. To avoid criticism of confounding effect of cell proliferation increased by 660-nm LED, % of cell population on and under the transwell was automatically counted through Olympus microscope software program and both value were sum. And then corrected number of invasion cells were calculated by dividing the number of invasion cells by the total % of population. To enhance astrocyte migration, it was performed in both conditions with or without chemoattractant and compared between each group. As a normal condition, a plate was filled with DMEM + both above and under a transwell, but in a chemoattractant condition, DMEM without FBS (DMEM and 1% P/S) was above a transwell and DMEM + (DMEM, 1% P/S and 10% FBS) was under a transwell.

#### Epifluorescence assessment

Cells were fixed in 100% MeOH and permeabilized with 0.3% Triton X-100 (Sigma-Aldrich) in 1 × PBS (Biosesang). After blocking in 10% bovine serum albumin (BioShop, Burlington, ON, Canada), cells were incubated overnight at 4 °C with the following primary antibodies: anti-glial fibrillary acidic protein (GFAP; MAB360, EMD Millipore, Burlington, MA, USA) and anti-DBN (ab178408, Abcam, Cambridge, UK). After the primary antibody reaction was completed, cells were washed with 1 × PBS and then incubated with Alexa488 and Alexa555 conjugated secondary antibodies. (A11001 and A21428, Invitrogen, Carlsbad, CA, USA) for 1.5 h at room temperature. Immunostained cells were washed three times with 1 × PBS for 5 min each, and then mounted with 4′,6-diamidino-2-phenylindole (DAPI)-containing VECTASHIELD anti-fade mounting medium (H-1200, Vector Laboratories, Burlingame, CA, USA). To obtain representative images, cells were observed with a confocal microscope (FV31-S, Olympus, Tokyo, Japan).

#### Statistical analyses

Statistical analyses were performed using the Prism v7.0 software (GraphPad, La Jolla, CA, USA). All experimental and control data are presented as means ± standard errors of the mean. The Shapiro–Wilk normality test was used to determine whether the data followed a Gaussian distribution; when this assumption was met, we performed two-tailed unpaired *t*-tests. One-way analysis of variance (ANOVA) was performed for multiple comparisons, followed by Tukey’s test, while Kruskal–Wallis test and Dunn’s multiple comparisons test were performed when the data don’t followed a Gaussian distribution. To investigate astrocyte processes, ROIs from the nucleus to the cell edge were captured and measured. Finally, cells were counted manually to analyze the expression patterns of DBN-associated proteins. Image analysis was evaluated using the ImageJ software (National Institutes of Health, Bethesda, MD, USA).

## Supplementary Information

Supplementary Figure S1.

## Data Availability

The datasets used and/or analysed during the current study available from the corresponding author on reasonable request.
