# Supplementary material for: Effects of 660-nm LED photobiomodulation on drebrin expression pattern and astrocyte migration
Source: Sci Rep. 2023 Apr 17;13:6220. doi: 10.1038/s41598-023-33469-5 (PMC10110518; doi:10.1038/s41598-023-33469-5)

**Effects of 660-nm LED photobiomodulation on drebrin expression pattern and astrocyte migration**

Sung Ryeong Yoon^1, 2^, So-Young Chang ^3^, Min Young Lee ^3, 4, *^ and Jin-Chul Ahn ^1, 2, 4, *^

^1^ Department of Medical Science, Graduate School of Medicine, Dankook University, Cheonan 31116, Republic of Korea.

^2^ Medical Laser Research Center, College of Medicine, Dankook University, Cheonan 31116, Republic of Korea.

^3^ Department of Otolaryngology-Head &Neck Surgery, College of Medicine, Dankook University, Cheonan 31116, Republic of Korea.

^4^ Beckman Laser Institute Korea, College of Medicine, Dankook University, Cheonan 31116, Republic of Korea.

Sung Ryeong Yoon: [dbstjdfud12@gmail.com](mailto:dbstjdfud12@gmail.com); So-Young Chang: so4040@hanmail.net

^*^Address all correspondence to Min Young Lee and Jin-Chul Ahn, [eyeglass210@gmail.com](mailto:eyeglass210@gmail.com); jcahn@dankook.ac.kr

**Supplementary Figure 1. Representative images and table for cell population on the transwell.** (A) Representative images show cells on the transwell that were not migrated (left) and images automatically analyzed by the Olympus microscope software program (right). Scale bar = 100 $\mu$m. (B) A table represents the number of non-migrated cells on transwell within each group (n = 3).


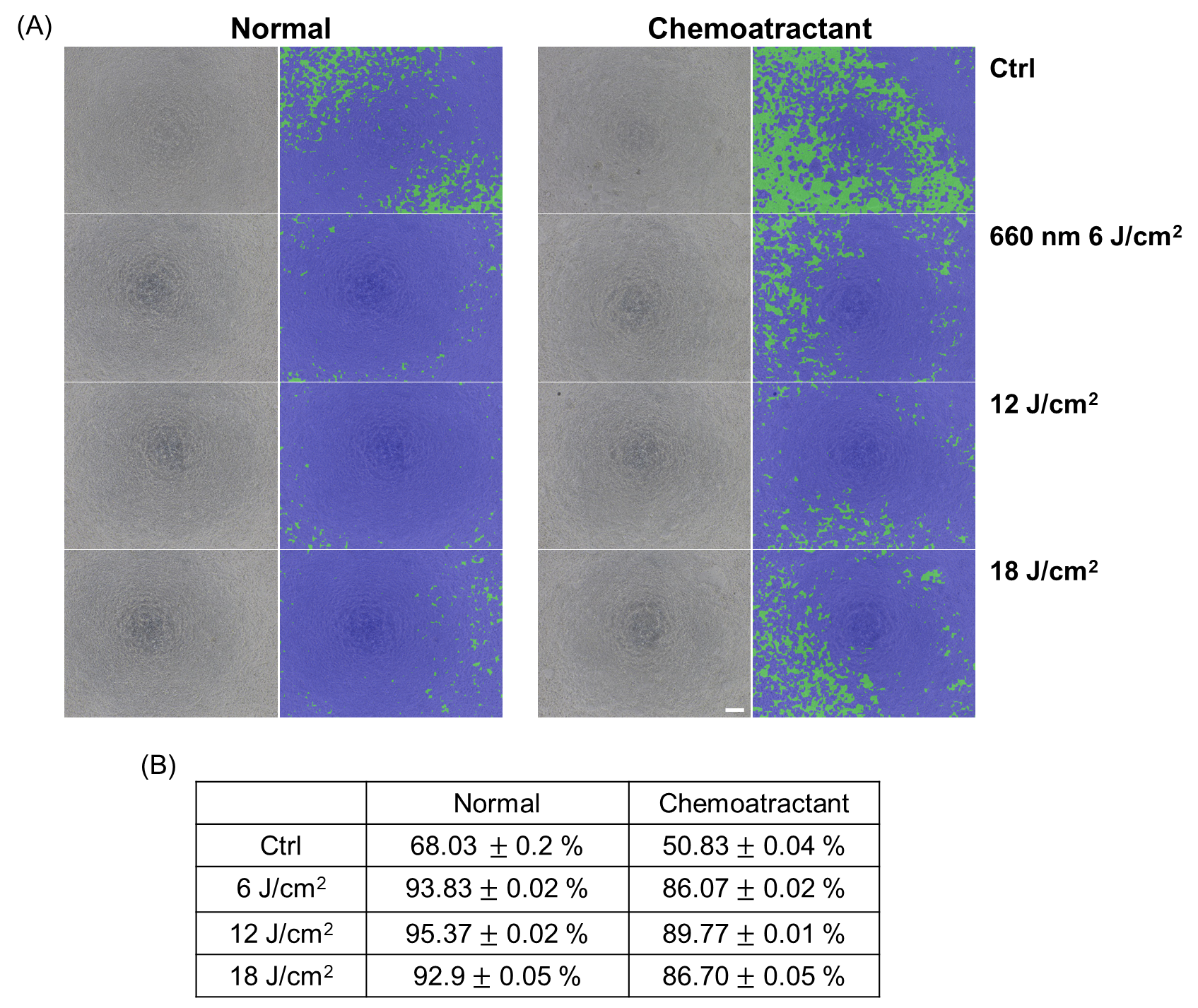

Supplement: Supplementary file 1 — Supplementary Figure S1. [file 41598_2023_33469_MOESM1_ESM.docx]
